# Depressive, Anxiety Symptom Frequency and Related Factors Among Prisoners During the COVID-19 Pandemic in Northeastern Ethiopia, a Cross-Sectional Study

**DOI:** 10.3389/fpsyt.2022.820015

**Published:** 2022-05-17

**Authors:** Mengesha Birkie, Mogesie Necho, Mekonnen Tsehay, Habtam Gelaye, Abeba Beyene, Asmare Belete, Amare Asmamaw, Zemenu Tadesse Tessema, Kassahun Bogale, Metadel Adane

**Affiliations:** ^1^Department of Psychiatry, College of Medicine and Health Sciences, Injibara University, Injibara, Ethiopia; ^2^Department of Psychiatry, College of Medicine and Health Sciences, Wollo University, Dessie, Ethiopia; ^3^Department of Epidemiology and Biostatistics, Institute of Public Health, College of Medicine and Health Sciences, University of Gondar, Gondar, Ethiopia; ^4^Department of Pharmacy, College of Medicine and Health Sciences, Dessie, Ethiopia; ^5^Department of Environmental Health, College of Medicine and Health Science, Wollo University, Dessie, Ethiopia

**Keywords:** depression, anxiety, prisoner, factors, Ethiopia

## Abstract

**Background:**

Among the more than 10 million people imprisoned around the world, the rate of mental illness is higher than among the general population for various reasons. Although rates of mental illnesses such as depression and anxiety in this population may have changed as a response to the coronavirus disease (COVID-19) outbreak and other factors, to our knowledge, no related studies have been conducted related to depression and anxiety in this population during the pandemic. Therefore, this study aimed to assess depression, anxiety, and associated factors among Dessie City prisoners during the 2020 COVID-19 outbreak.

**Methods:**

An institution-based cross-sectional survey was conducted in October 2020. A total of 420 prisoners were selected *via* a systematic sampling technique. PHQ-9 depression scale, generalized anxiety disorder-7 questionnaire, Oslo 3-item social support scale, insomnia severity index, and Brief COPE scale were used. Data were entered by using Epi-Data version 3.1 and finally exported to Statistical Package for Social Science Software version 21 for analysis. We fitted a multiple binary logistic regression model. Finally, an adjusted odds ratio with 95% confidence interval was reported and factors with a *p*-value < 0.05 were considered as significant for depression and anxiety.

**Results:**

This study showed that 279 (66.4%) of imprisoned people had major depressive disorder with 95% CI of (61.4, 70.6), while 281 (66.9) had generalized anxiety disorder with 95% CI of (61.9, 71.9).

**Conclusion:**

In this study, the overall prevalence of depression and anxiety was significantly high, and was related to a number of factors including COVID-19. Therefore, designing and implementing strategies for COVID-19 prevention and control in prisons is highly recommended to reduce mental health problems among prisoners.

## Background

Coronavirus disease (COVID-19), similar to severe acute respiratory syndrome (SARS). Bats are thought to have been the initial route of transmission but we have an ongoing pandemic because it is being widely transmitted from person to person currently (1, 2). Study of person-to-person transmission has suggested that on average, every case of COVID-19 will create up to four new cases (3). The rate of cases reported after 17 January 2020 increased 21-fold in comparison to the first half of January 2020 (4). The world health organization publicly declared a global pandemic on March 11, 2020 (5). This pandemic disease rapidly spread around the globe, infecting and killing many people (6).

Mental illness is a condition characterized by significant disturbance in cognition, emotional regulation, and behavioral functioning (7). Mental health problems such as depression, anxiety, and others are a common response to the COVID-19 outbreak (3).

Worldwide, more than 10 million people were imprisoned, of whom over 7 million were from low- and middle-income countries (LMIC; 8, 9). The rate of mental disorders among prisoners is at least five times higher than in the general population, (10–12). The overall prevalence of mental disorders was 65% among prisoners (13). In comparison, depression and anxiety among the general population were 23.8 and 13.1%, respectively, (14).

Prisoners are highly vulnerable to a mental disorder as compared to the wider community (15), a finding supported by a study done in the United Kingdom that found major depressive disorder was 61.7% and of anxiety 53.3% among incarcerated women (16). In addition, a study of young offenders in Chile found 22% had depression and 24% had an anxiety disorder (17). One recently conducted study among New Zealand prisoners revealed that nearly a third (32%) had a mood disorder (depression), while 30% had an anxiety disorder (18). During the COVID-19 outbreak, mental health problems such as depression and anxiety disorder in various populations have been reported as 48.3 and 22.6%, respectively, (7, 19). Other locations of mental health findings among the general population during the COVID-19 outbreak include Hong Kong where 19% had depression, while 14% had anxiety (20), the United States, which revealed 30.8 and 24.9% rates of anxiety and depression, respectively, (21) Canada, where generalized anxiety disorder and major depressive disorder were 47.2 and 44.1%, respectively, (22), Portugal and Brazil, where 71.3% had anxiety and 24.7% had depression (23). One systematic review and meta-analysis study during the COVID-19 outbreak reported that the prevalence of depression and anxiety was 33.7 and 31.9%, respectively, (24). Furthermore, in Iranian prisoners, the rate of major depression was 44%, although anxiety disorder had the highest prevalence at 56.3% (25).

The prevalence of depression and anxiety among prisoners being very high is supported by a number of studies including one conducted in Nigeria where 35% had depression (26), while 37% suffered similarly in another study in southern Nigeria (27). In another study conducted in Ethiopia prisoners, the prevalence of depression was 43.8% in North West Amhara regional state (28); in Bahir Dar, 45.5% (29); in southern Ethiopia 56.4% (30); and 44% in Debre Birhan (31). Furthermore, prisoners in Jimma reported 41.9 and 36.1% rates of depression and anxiety, respectively, (32, 33).

Some factors that have been significantly associated with mental illness (depression and anxiety) among prisoners are socio-demographic factors (4, 34), a long period of imprisonment, social activities, gender, and marital status (13). Moreover, being female (14, 15), divorced, or having an underlying chronic disease (14), being widowed, educated at college or university level, or having a sentence length of 5–10 years were risk factors for depression (31).

Risk factors for depression were also found to be having a lifetime substance use, being between 21 and 25 years of age, having poor social support (32), and being a smoker (33), level of social support (28), being sentenced to more than 5 years or 1–5 years (29), having both a chronic disease and history of khat use (26, 30, 32). The COVID-19 pandemic is still a devastating problem all over the world that affects all people in their day-to-day activities; in one Ethiopian study, 44.4% of subjects reported the pandemic was causing mild to moderate psychological problem (35). Although there has been substantial attention paid to identifying people infected with coronavirus, identification of the mental health care needs of people impacted by this pandemic has been relatively neglected (36).

Earlier research showed that following COVID-19 news frequently (5), this frequent media exposure may cause distress and increases the vulnerability to the psychological impact of COVID-19 (5, 37–39). A researcher of this study, we know that people living in persons, subjected to different forms of mental and psychological problems specially, during emergency crisis like COVID-19, and the method they cope may not be adaptive.

To our knowledge, no studies have been conducted related to depressive, anxiety symptom frequency and related factors among prisoners during the COVID-19 pandemic in Dessie, Amhara Region, northeastern Ethiopia. Therefore, this study aimed to delivering appropriate messages, equipping them with updated information, reducing the risk of psychological distress, finally improving the psychosocial health of people living in person in Dessie town.

## Materials and Methods

### Study Design, Period, and Setting

An institution-based cross-sectional study was conducted in October, 2020. Dessie is the capital city of South Wollo Zone in the Amhara National Regional State. It is found in northeastern Ethiopia, 400 km north of Addis Ababa. Dessie is the third-largest metropolitan city next to Bahirdar and Gondar in the Amhara region; data from the 2016–2017 report of the South Wollo Zone statistics office estimated Dessie’s population was more than 350,000. During data collection for this study in October, 2020. The number of prisoners in Ethiopia is 63,792 of them 14,489 found in Amhara region (40). Specifically in Dessie’s correctional facility had a total of 1,550 prisoners who either had a court decision or were waiting for a court decision.

### Study Participants

The study participants were all prisoners in Dessie who were available during the period of data collection. The optimal size of the sample was calculated using a single population proportion formula, by taking 50% prevalence with a 5% margin of error and 95% confidence interval of certainty (alpha = 0.05) with a 10% non-response rate. Based on these assumptions, the sample size for the study was 423; of that number, a total of 420 prisoners were involved in the study. Systematic random sampling technique was employed to recruit study participants. The first study unit was selected randomly between first and *k*th (*k* = 4) study participants and every fourth individual was interviewed; when someone refused to participate in the study, the next eligible respondent was interviewed. Any individual whose age was under 18 years, who had been diagnosed with a mental illness or was unable to hear and speak was excluded from the study.

### Operational Definitions

• Level of depression: A total of patient health questionnaire (PHQ-9) score of 0–4, 5–9, 10–14, 15–19, or 20–27 was considered as no depression, mild depression, moderate depression, moderately severe depression, and severe depression, respectively, (41).
• Major depressive disorder: A score of ≥10 on the PHQ-9 scale was considered as a major depressive disorder (41).
• Anxiety: A score on generalized anxiety disorder-7 scale (GADs-7) of 8–10, 11–14, or 15–21 was considered as mild, moderate and severe, respectively, (42, 43).
• General anxiety disorder: A score of >10 on the GADs-7 scale was considered as a general anxiety disorder (42–44).
• Poor social support: The sum of the raw scores being 3–8 (45).
• Intermediate social support: The sum of the raw scores being 9–11 (45).
• Strong social support: The sum of the raw scores being 12–14 (45).
• Level of coping strategies: The sum of the raw scores being 4–13, 14–16, or 17–20 was considered as having low, moderate, and high coping strategies, respectively, (46).
• Level of insomnia: A score on ISI scale of 0–7, 8–14, 15–21, or 22–28 was considered as no clinical sign of insomnia, having sub-threshold insomnia, having clinical moderately severe insomnia, and having clinically severe insomnia, respectively, (47).
• Having low resilient coping: From brief resilient coping scale, a score of 4–13 points (48).
• Having moderate coping: From brief resilient coping scale, a score of 14–16 points (48).
• Having high coping: From brief resilient coping scale, a score of 17–20 points (48).
• Chronic physical illness: In this study, defined presence of one or more chronic medical, surgical, or neurological problems.
• Substance use: In this study, defined as ever using at least one specified substance before imprisonment.
• Ever substance user: Having ever used the specified substance even once in the participant’s lifetime.
• Current substance user: Having used a specified substance in the last 30 days.

### Data Collection Procedures

The questionnaires were prepared in English, then translated into the Amharic language to be used to collect the data. PHQ-9 scale was used to assess the prevalence of major depressive disorder. PHQ-9 items showed good internal (Cronbach’s alpha = 0.81) and test re-test reliability (interclass correlation coefficient = 0.92; sensitivity = 86% and specificity = 67%). The PHQ-9 appears to be a reliable and valid instrument that used to diagnose major depressive disorders in Ethiopia (41).

Generalized anxiety disorder-7 scale was used to assess the presence of anxiety disorder. It is a 7-point item questionnaire and each has a 4-point Likert scale, which ranges from 0 to 3 where 0 means not at all, 1 means several days, 2 means more than half the days, and 3 means nearly every day. It has a total score ranging from 0 to 21. When screening for anxiety disorders, a recommended cut-point for further evaluation is a score of 10 or greater (42, 43, 49).

Oslo 3-items social support scale (OSS-3) provided a brief measure of social support. It was validated in Ethiopia with sensitivity and specificity of 84.2 and 82.7%, respectively, (45). Insomnia severity index (ISI) contains 7 questions and answers on a 5-point scale to rate sleep problems. The sum of the answers provides a level of insomnia score: A score 0–7, 8–14, 15–21, and 22–28 were considered as no clinical signs of insomnia, having sub-threshold insomnia, having clinical moderately severe insomnia, and having clinically severe insomnia, respectively, (47).

We also used the Brief COPE (50) scale to measure coping strategies. The brief COPE is a shortened version of the COPE scale and has been used widely, including in LMICs. The Brief COPE scale was found to have reasonable reliability and validity (51). The Brief COPE scale was originally developed to measure coping with any stressor (50, 52). The scale has 20 items that assess the degree to which a respondent utilizes a specific coping strategy. The 20 items have been categorized into 3 levels of coping strategies: low, moderate, and high (46, 48).

### Data Collection and Data Quality Assurance

Primary data were collected from individual prisoners by using a structured questionnaire, PHQ-9, GADs-7, OSS-3, ISI, and Brief COPE. Data were collected by two BSc graduate assistant psychiatry nurses. The principal investigator was involved in overall control of data collection. Supervisors were given 1 day of training by the principal investigator on the study instrument, consent form, how to maintain confidentiality, and data collection procedures based on the tools processed. To assure the data quality, assessment tools that were internationally recognized and had been validated in Ethiopia were used, including PHQ-9, GADs-7, OSS-3, ISI, Brief COPE, and a socio-demographic questionnaire. An Amharic version of the questionnaire was used for the interview. Regular supervision was given by the principal investigator to ensure that all necessary data were properly collected. Each day during data collection, questionnaires were checked for completeness and consistency. Data cleaning and editing were made before actual data analysis. A pre-test was conducted on 5% of the sample among prisoners from the city of Kombolcha. After pre-testing, corrections to wording on the questionnaire that the participants found ambiguous were made. Data that were collected in the pre-test were not included in the analysis as part of the main study.

### Data Analysis Procedures and Interpretation

After collection was completed, data were entered into a computer using Epi-Data version 3.1 statistical software. Then the data were exported into Statistical Packages for Social Sciences (SPSS) version 21 for Windows. Data cleaning and editing were done before actual data analysis. Descriptive statistics for frequencies, means and standard deviation were calculated to summarize the dependent and independent variables. Both bivariate and multivariable logistic regressions were used to determine the association of socio-demographic factors and clinical independent variables with depression and anxiety. First, each independent variable was entered into bivariate analysis, one by one. Then, variables associated with depression and anxiety with a *p*-value of less than 0.25 on bivariate analysis were entered into multiple logistic regression all together to control confounders. Finally, variables with a *p*-value of <0.05 on multivariable regression were considered as predictors of depression and anxiety. Results were presented using frequency distribution, percentages, tables, and figures.

## Results

### Socio-Demographic Characteristics

In this study, from a total of 423 participants, 420 completed the interview, for a response rate of 99.2%. The mean age of the respondents was 29.79 year with SD ± 8.29 and range was 18–62 year. The majority of respondents 333 (79.3%), 338 (80.5%), 292 (69.5%), and 292 (69.5%) were male, Amhara, living with family before being arrested and worried about being infected with COVID 19, respectively. However, more than half 216 (51.4%), 223 (53.1%), and 245 (58.3%) of participants were married, of Orthodox religion, and knew their court decision, respectively. One hundred thirty-six (32.4%) of the participants had a secondary-education level. Two hundred five (48.8%) had a high level of fear for getting COVID-19 infection. Likewise, 136 (38.6%) of participants, who showed symptoms resembling COVID-19, had high level of worry about being infected by COVID-19,eventhough they were not tested (Table 1).

**TABLE 1:** Socio-demographic characteristics of study participants among Dessie town prisoners in Dessie Amhara region, Northeast Ethiopia, October 2020; (*n* = 420).

Characters		Frequency (%)
Age	18–25 26–33 34–41 >or = 42	148(35.2%) 142(33.8%) 103(24.5%) 27(6.4%)
Sex	Male Female	333(79.3%) 87(20.7%)
Marital status	Married Single Divorced Widowed	216(51.4%) 177(42.1%) 14(3.3%) 13(3.1%)
Ethnicity	Amhara Oromo Tigre Others (afar, gurage, and kebata)	338(80.5%) 46(11.0%) 30(7.1%) 6(1.4%)
Religion	Orthodox Muslim Protestant Others (Waqifeta and Joba witness)	223(53.1%) 156(37.1%) 25(6.0%) 16(3.8%)
Educational status	Illiterate Primary Secondary College and above	77(18.3%) 97(23.1%) 136(32.4%) 110(26.2%)
Occupational status	“Governmental employee” Farmer Merchant “Housewife” “Daily laborer” Others (Student and driver)	78(18.6%) 97(23.1%) 94(22.4%) 17(4.0%) 58(13.8%) 76(18.1%)
Living residency before arrested	Living alone With family Social apartment	115(27.4%) 292(69.5%) 13(3.1%)
Worried about being infected with COVID-19	Yes No	292(69.5%) 128(30.5%)
Level of fear for oneself and one’s family getting infected	High Moderate Low No	205(48.8%) 115(27.4%) 29(6.9%) 71(16.9%)
Level of worry about getting infected while showing symptoms associated with COVID-19	High Moderate Low None	162(38.6%) 121(28.8%) 38(9.0%) 99(23.6%)
Type of crime done by the respondents	Robber Murder Conflict Sexual Abuse Others (Corruption and political issue)	98(23.3%) 118(28.1%) 82(19.5%) 64(15.2%) 58(13.8%)
Number of stay in the prison	< or = 1 year 1–2 year 3–4 year >or = 5 year	203(48.3%) 121(28.8%) 41(9.8%) 55(13.1%)
Knowing court decision	Yes No	245(58.3%) 175(41.7%)
History of arrested before this time	Yes No	43(10.2%) 377(89.8%)

Of the total 420 respondents, 104 (24.8%) had a history of chronic disease, including hypertension 26 (6.2%), diabetes mellitus 31 (7.4%), cardiovascular disease 11 (2.6%), HIV 28 (6.7%), or other (asthma, cancer, and neurologic disorders) 8 (1.9%). Those who reported ever being a substance user numbered 86 (20.5%), including 21 (5%) alcohol, 18 (4.3%) khat, 6 (1.4%) cigarettes, and 41 (9.8%) had used two or more substances at least once. One hundred forty-four (34.3%) had clinical moderately severe insomnia and the majority 286 (68.1) had poor social support. Similarly, 176 (41.9%) had low level of coping mechanisms (Tables 2, 3).

**TABLE 2:** History of Chronic illness and substance use behavior during COVID-19 pandemic of study participants among Dessie town prisoners in Dessie Amhara region, Northeast Ethiopia, October 2020; (*n* = 420).

Characters		Frequency (%)
Chronic disease history of the participants	Yes No	104(24.8%) 316(75.2%)
Type of chronic disease reported by the respondents	Hypertension Diabetes mellitus Cardio-Vascular Hive Other* No history of chronic illness	26(6.2%) 31(7.4%) 11(2.6%) 28(6.7%) 8(1.9%) 316(75.2%)
Ever substance use	Yes No	86(20.5%) 334(79.5%)
Type of substance use	Alcohol Kat Cigarette two or more Substance user Never use at all	21(5.0%) 18(4.3%) 6(1.4%) 41(9.8%) 334(79.5%)
Current substance use	Yes No Never use in their life	13(3.1%) 73(17.4%) 334(79.5%)

*Chronic illness (Asthma, Cancer, and Neurologic disorder).

**TABLE 3:** Level of severity for insomnia social support, depression, anxiety, and copping mechanism during COVID-19 pandemic among Dessie town prisoners, in Dessie Amhara region, Northeast Ethiopia, October 2020; (*n* = 420).

Characters		Frequency (%)
Level of insomnia severity	No clinical sign of insomnia Having sub threshold insomnia Clinically having moderately sever insomnia Clinically having sever insomnia	60(14.3%) 134(31.9%) 144(34.3%) 82(19.5%)
Social support	Poor social support Moderate social support Strong social support	286(68.1%) 92(21.9%) 42(10.0%)
Level of depression severity	No depression Having mild depression Having moderate depression Having moderately severe depression Having severe depression	60(14.3%) 58(13.8%) 121(28.8%) 133(31.7%) 48(11.4%)
Level of anxiety severity	Minimal anxiety Mild anxiety Moderate anxiety Sever anxiety	63(15.0%) 56(13.3%) 129(30.7%) 172(41.0%)
Level of coping mechanism	Having low coping mechanism Having moderate coping mechanism Having high coping mechanism	176(41.9%) 111(26.4%) 133(31.7%)

### Prevalence of Major Depressive and Generalized Anxiety Disorder Among Study Participants

**FIGURE 1:**
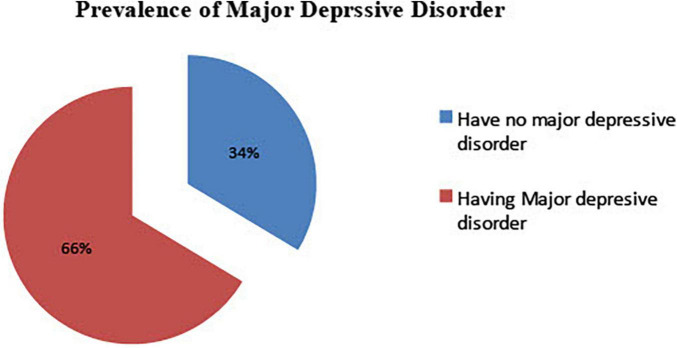
Major depressive disorder during COVID-19 pandemic, among Dessie town prisoners, in Dessie Amhara region, Northeast Ethiopia, October 2020; (*n* = 420).

**FIGURE 2:**
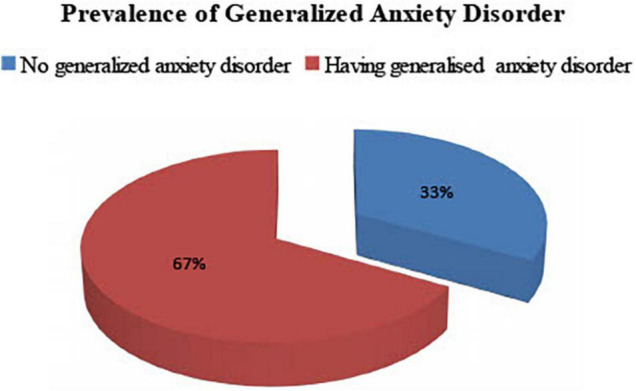
Generalized anxiety disorder during COVID-19 pandemic, among Dessie town prisoners, in Dessie Amhara region, Northeast Ethiopia, October 2020; (*n* = 420).

Of the 420 study participants, 279 (66.4%) had major depressive disorder with 95% CI of (61.4, 70.6). At least 61.4% and at most 70.6% of prisoners (lower and upper 95% CI) were found to have a major depressive disorder. Likewise, 281 (66.9) had generalized anxiety disorder with a 95% CI of (61.9, 71.9). At least 61.9% and at most 71.9% of prisoners (lower and upper 95% CI) were found with generalized anxiety disorder (Figures 1, 2).

### Socio-Demographic Risk Factors for Major Depressive Disorder on First Model Analysis

On binary analysis, the variables having *p* < 0.25 were age, ethnicity, occupation, residence before being arrested, level of fear for self, and one’s family getting infected with COVID-19, level of worry about being infected while showing symptoms associated with COVID-19, length of stay in the prison, having a history of previous arrest, insomnia, social support, level of coping mechanism, having a chronic disease, type of chronic disease, and having ever been a substance user. However, sex, marital status, ethnicity, religion, educational status, being worried about being infected with COVID-19, type of crime done, and knowing one’s court decision didn’t reveal an association with major depressive disorder on the crude odds ratio.

### Factors Associated With Major Depressive Disorder

On multiple logistic regression, variables significantly associated with major depressive disorder were found. Those having no clinical sign of insomnia were 92% less likely to have a major depressive disorder [AOR = 95% CI: 0.08 (0.03, 0.21)] as compared to those having clinically severe insomnia. Similarly, those having clinical moderately severe insomnia were 82% less likely to have a major depressive disorder [AOR = 95% CI: 0.18 (0.08, 0.42)] as compared to those having clinically severe insomnia. Also, those who had moderate social support or strong social support were 51 and 64% less likely to have a major depressive disorder [AOR = 95% CI: 0.49 (0.27, 0.88) and 0.36 (0.16, 0.80)], respectively, as compared to those having poor social support. Those having a history of diabetes mellitus were 82% less likely to have a major depressive disorder [AOR = 95% CI: 0.18 (0.04, 0.76)] compared to those having HIV. Lastly, those participants who had history of substance use were all most two times more likely to have major depressive disorder [AOR = 95% CI: 1.89 (1.07, 3.34)] as compared to those who had no (Tables 4, 5).

**TABLE 4:** Multiple logistic regression: Factors associated independently with major depressive disorder during COVID-19 pandemic among Dessie town prisoners, in Dessie Amhara region, Northeast Ethiopia, October 2020; (*n* = 420).

Characters		Major depressive disorder		COR (95%CI)	AOR (95%CI)
		
		No	Yes		
Age	18–25 26–33 34–41 >or = 42	55(39.0%) 55(39.0%) 24(17.0%) 7(5.0%)	93(33.3%) 87(31.2%) 79(28.3%) 20(7.2%)	0.59(0.23,1.49) 0.55(0.22,1.39) 1.15(0.43,3.05) 1	0.83(0.26,2.64) 0.59(0.19,1.84) 1.16(0.35,3.82) 1
Ethnicity	Amhara Oromo Tigre Others	116(82.3%) 12(8.5%) 10(7.1%) 3(2.1%)	222(79.6%) 34(12.2%) 20(7.2%) 3(1.1%)	1.91(0.38. 0.63) 2.83(0.50,15.98) 2.00(0.34,11.75) 1	2.40(0.21,27.50) 3.06(0.24,38.40) 3.27(0.25,41.57) 1
Occupational status	Governmental employee Farmer Merchant Housewife Daily laborer’ Others	31(22.0%) 30(21.3%) 32(22.7%) 6(4.3%) 11(7.8%) 31(22.0%)	47(16.8%) 67(24.0%) 62(22.2%) 11(3.9%) 47(16.8%) 45(16.1%)	1.04(0.54,1.98) 1 1.53(0.82,2.88) 1.33(0.71,2.49) 0.1.26(0.42,3.77) 2.94(1.32,6.55)	0.93(0.42,2.03) 1 1.09(0.51,2.34) 1.66(0.40,6.85) 2.65(1.02,6.89) 1.10(0.49,2.43)
Residence before arrested	Living alone With Family Social apartment	43(30.5%) 91(64.5%) 7(5.0%)	72(25.8%) 201(72.0%) 6(2.2%)	1 1.95(0.61,6.19) 2.57(0.84,7.88)	1 1.61(0.93,2.79) 0.45(0.11,1.83)
Level of fear for oneself and one’s family getting infected	High Moderate Low No	55(39.0%) 53(37.6%) 9(6.4%) 24(17.0%)	150(53.8% 62(22.2%) 20(7.2%) 47(16.8%)	1 1.39(0.77,2.48) 0.59(0.32,1.10) 1.13(0.44,2.87)	1 0.62(0.33,1.15) 1.13(0.39,3.23) 2.13(0.86,5.26)
Level of worry about getting infected and when showing symptoms associated with COVID-19	High Moderate Low Non	49(34.8%) 37(26.2%) 10(7.1%) 45(31.9%)	113(40.5%) 84(30.1%) 28(10.0%) 54(19.4%)	1 1.92(1.14,3.22) 1.89(1.08,3.29) 2.33(1.02,5.31)	1 1.21(0.65,2.24) 2.24(0.85,5.91) 0.48(0.21,1.07)
Stay in the prison	<or = 1 year 1–2 year 3–4 year >or = 5 year	63(44.7%) 55(39.0%) 8(5.7%) 15(10.6%)	140(50.2%) 66(23.7%) 33(11.8%) 40(14.3%)	1 0.83(0.42,1.61) 0.45(0.22,0.90) 1.54(0.58,4.09)	1 0.61(0.35,1.06) 1.72(0.67,4.43) 1.64(0.75,3.62)
History of arrested before this time	Yes No	19(13.5%) 122(86.5%)	24(8.6%) 255(91.4%)	1 0.60(0.31,1.14)	1 1.70(0.77,3.76)
Social support	Poor social support Moderate social support Strong social support	79(56.0%) 41(29.1%) 21(14.9%)	207(74.2%) 51(18.3%) 21(7.5%)	1 2.62(1.35,5.06) 1.24(0.59,2.58)	1 0.49(0.27,0.88)* 0.36(0.16,0.80)*
Level of coping mechanism	Having low copper Having moderate copper Having high copper	67(47.5%) 43(30.5%) 31(22.0%)	109(39.1%) 68(24.4%) 102(36.6%)	1 0.49(0.29,0.82) 0.481(0.27,0.83)	1 1.00(0.55,1.80) 1.74(0.95,3.17)
Chronic disease history of the participants	Yes No	24(17.0%) 117(83.0%)	80(28.7%) 199(71.3%)	1 1.96(1.17,3.26)	1 0.36(0.12,1.04)
Type of chronic disease	Hypertension Diabetes mellitus Cardio-Vascular Hive Other No history of Medical illness	6(4.3%) 13(9.2%) 3(2.1%) 5(3.5%) 3(2.1%) 111(78.7%)	20(7.2%) 18(6.5%) 8(2.9%) 23(8.2%) 5(1.8%) 205(73.5%)	0.72(0.19,2.73) 0.30(0.09,1.00) 0.58(0.11,2.99) 1 1.08(0.18,6.57) 0.36(0.06,2.03)	1.46(0.27,7.78) 0.18(0.04,0.76)* 0.37(0.05,2.48) 1 0.44(0.05,3.76) 0.78(0.18,3.37)

*A significant only by bivariable logistic regression, variables that have p-value less than 0.05 in multivariable logistic regression were considered as significant.

**TABLE 5:** Multiple logistic regression: Factors associated with major depressive disorder level of severity of insomnia, social support, copping mechanism, chronic disease, and substance use with major-depressive disorder during COVID-19 pandemic among Dessie town prisoners, in Dessie Amhara region, Northeast Ethiopia, October 2020; (*n* = 420).

	Characters	Major depressive disorder		COR (95%CI)	AOR (95%CI)
		
		No	Yes		
Level of insomnia severity	No clinical sign of insomnia Having sub threshold insomnia Clinically having moderately sever insomnia Clinically having sever insomnia	37(26.2%) 58(41.1%) 35(24.8%) 11(7.8%)	23(8.2%) 76(27.2%) 109(39.1%) 71(25.4%)	0.09(0.04,0.21) 0.20(0.09,0.41) 0.48(0.23,1.01) 1	0.08(0.03,0.21)* 0.18(0.08,0.42)* 0.51(0.22,1.18) 1
Social support	Poor social support Moderate social support Strong social support	79(56.0%) 41(29.1%) 21(14.9%)	207(74.2%) 51(18.3%) 21(7.5%)	1 2.62(1.35,5.06) 1.24(0.59,2.58)	1 0.49(0.27,0.88)* 0.36(0.16,0.80)*
Level of coping mechanism	Having low copper Having moderate copper Having high copper	67(47.5%) 43(30.5%) 31(22.0%)	109(39.1%) 68(24.4%) 102(36.6%)	1 0.49(0.29,0.82) 0.481(0.27,0.83)	1 1.00(0.55,1.80) 1.74(0.95,3.17)
Chronic disease history of the participants	Yes No	24(17.0%) 117(83.0%)	80(28.7%) 199(71.3%)	1 1.96(1.17,3.26)	1 0.36(0.12,1.04)
Type of chronic disease	Hypertension Diabetes mellitus Cardio-Vascular Hive Other No history of Medical illness	6(4.3%) 13(9.2%) 3(2.1%) 5(3.5%) 3(2.1%) 111(78.7%)	20(7.2%) 18(6.5%) 8(2.9%) 23(8.2%) 5(1.8%) 205(73.5%)	0.72(0.19,2.73) 0.30(0.09,1.00) 0.58(0.11,2.99) 1 1.08(0.18,6.57) 0.36(0.06,2.03)	1.46(0.27,7.78) 0.18(0.04,0.76)* 0.37(0.05,2.48) 1 0.44(0.05,3.76) 0.78(0.18,3.37)
Ever substance use	Yes No	36(25.5%) 105(74.5%)	50(17.9%) 229(82.1%)	0.63(0.39,1.03) 1	1.89(1.07,3.34)* 1

*A significant only by bivariable logistic regression, variables that have p-value less than 0.05 in multivariable logistic regression were considered as significant.

### Socio-Demographic Factors for Generalized Anxiety Disorder on Bivariate Analysis

On bivariate analysis for generalized anxiety disorder, factors with a *p*-value of ≤0.25, were age, sex, occupation, level of fear for oneself and one’s family of getting infected with COVID-19, level of worry while showing symptoms associated with COVID-19, type of crime done, length of prison stay, level of insomnia, degree of social support, level of coping mechanism, chronic disease, type of chronic disease, and ever having used a substance. However, marital status, ethnicity, religion, educational status, place of residence before being arrested, worried about being infected with COVID-19, level of fear for oneself and one’s family of getting infected, knowing one’s court decision, a history of arrest before this instance and being a current substance user were not eligible in the final model.

### Factors Independently Associated With Generalized Anxiety Disorder

On multiple logistic regression, study participants’ age was shown to be significantly associated with generalized anxiety disorder; those with ages from 18–25 to 26–33 were 81 and 82% less likely to have generalized anxiety disorder [AOR = 95% CI: 0.19 (0.05, 0.72) and 0.18 (0.05, 0.68)], respectively, than those 42 years old or older. However, being a merchant was found to increase the chance of developing generalized anxiety disorder among the participants more than two times [AOR = 95% CI: 2.08 (1.02, 4.25)] as compared to being a farmer. Likewise, being a housewife increased the chance of, having generalized anxiety disorder more than eight times [AOR = 95% CI: 8.51 (1.49, 48.65)] as compared to being a farmer (Table 6).

**TABLE 6:** Multiple logistic regression: Factors associated with level of severity of insomnia, social support, copping mechanism, chronic disease, and substance use with generalized anxiety disorder during COVID-19 pandemic among Dessie town prisoners, in Dessie Amhara region, Northeast Ethiopia, October 2020; (*n* = 420).

	Characters	Major depressive disorder		COR (95%CI)	AOR (95%CI)
		
		No	Yes		
Age	18–25 26–33 34–41 >or = 42	55(39.0%) 55(39.0%) 24(17.0%) 7(5.0%)	93(33.3%) 87(31.2%) 79(28.3%) 20(7.2%)	0.28(0.09,0.86) 0.28(0.09,0.86) 0.54 (0.17,1.71) 1	0.19(0.05,0.72)* 0.18(0.05,0.68)* 0.34(0.09,1.31) 1
Sex	Male Female	116(82.3%) 25(17.7%)	217(77.8%) 62(22.2%)	0.58(0.33,0.99) 1	0.55(0.29,1.05) 1
Occupational status	Governmental employee Farmer Merchant Housewife Daily laborer’ Others	31(22.0%) 30(21.3%) 32(22.7%) 6(4.3%) 11(7.8%) 31(22.0%)	47(16.8%) 67(24.0%) 62(22.2%) 11(3.9%) 47(16.8%) 45(16.1%)	0.81(0.44,1.49) 1 1.40(0.76,2.57) 4.23(0.91,19.60) 1.61(0.78,3.32) 0.96(0.51,1.80)	1.14(0.56,2.35) 1 2.08(1.02,4.25)* 8.51(1.49,48.65)* 2.00(0.89,4.52) 1.42(0.68,2.96)
Level of fear for oneself and one’s family getting infected	High Moderate Low No	55(39.0%) 53(37.6%) 9(6.4%) 24(17.0%)	150(53.8% 62(22.2%) 20(7.2%) 47(16.8%)	1 1.15(0.64,2.04) 0.85(0.46,1.59) 1.13(0.44,2.87)	1 0.99(0.52,1.86) 0.92(0.32,2.66) 1.24(0.51,3.01)
Worry while showing symptoms associated with COVID-19	High Moderate Low Non	49(34.8%) 37(26.2%) 10(7.1%) 45(31.9%)	113(40.5%) 84(30.1%) 28(10.0%) 54(19.4%)	1.11(0.66,1.87) 1 1.66(0.94,2.93) 1.74(0.76,3.99)	1.87(1.05,3.35)* 1 2.10(0.85,5.18) 1.00(0.52,1.90)
Type of crime done	Robber Murder Conflict Sexual Abuse Others	30(21.3%) 42(29.8%) 27(19.1%) 20(14.2%) 22(15.6%)	68(24.4%) 76(27.2%) 55(19.7%) 44(15.8%) 36(12.9%)	1 1.20(0.61,2.36) 1.06(0.55,2.04) 1.56(0.76,3.21) 1.56(0.73,3.34)	1 1.00(0.50,2.00) 1.10(0.51,2.40) 1.29(0.55,3.06) 1.03(0.45,2.36)
Stay in the prison	<or = 1 year 1–2 year 3–4 year >or = 5 year	63(44.7%) 55(39.0%) 8(5.7%) 15(10.6%)	140(50.2%) 66(23.7%) 33(11.8%) 40(14.3%)	1 0.97(0.50,1.88) 0.66(0.33,1.33) 0.57(0.24,1.35)	1 1.89(1.01,3.55)* 2.30(0.88,6.04) 0.99(0.44,2.24)
Level of insomnia severity	No clinical sign of insomnia Having sub threshold insomnia Clinically having moderately severe insomnia Clinically having sever insomnia	37(26.2%) 58(41.1%) 35(24.8%) 11(7.8%)	23(8.2%) 76(27.2%) 109(39.1%) 71(25.4%)	0.08(0.03,0.19) 0.19(0.09,0.42) 0.29(0.13,0.65) 1	0.05(0.01,0.13)* 0.13(0.05,0.31)* 0.23(0.09,0.54)* 1
Social support	Poor social support Moderate social support Strong social support	79(56.0%) 41(29.1%) 21(14.9%)	207(74.2%) 51(18.3%) 21(7.5%)	1 2.53(1.31,4.88) 1.48(0.71,3.09)	1 0.57(0.32,1.03) 0.25(0.11,0.56)*
Level of coping mechanism	Having low copper Having moderate copper Having high copper	67(47.5%) 43(30.5%) 31(22.0%)	109(39.1%) 68(24.4%) 102(36.6%)	1 0.63(0.38,1.03) 0.71(0.41,1.23)	1 1.33(0.74,2.38) 1.40(0.76,2.57)
History of chronic disease	Yes No	24(17.0%) 117(83.0%)	80(28.7%) 199(71.3%)	1 1.90(1.14,3.17)	1 0.78(0.29,2.08)
Type of chronic disease	Hypertension Diabetes mellitus Cardio-Vascular Hive Other No history of Medical illness	6(4.3%) 13(9.2%) 3(2.1%) 5(3.5%) 3(2.1%) 111(78.7%)	20(7.2%) 18(6.5%) 8(2.9%) 23(8.2%) 5(1.8%) 205(73.5%)	0.41(0.11,1.44) 0.45(0.13,1.55) 0.97(0.16,5.98) 1 0.65(0.10,4.23) 0.40(0.14,1.08)	0.34(0.07,1.60) 0.37(0.08,1.58) 0.34(0.04,2.70) 1 0.54(0.06,4.71) 0.38(0.09,1.56)
Ever substance use	Yes No	36(25.5%) 105(74.5%)	50(17.9%) 229(82.1%)	1 1.35(0.80,2.28)	1 0.67(0.35,1.26)

*A significant only by bivariable logistic regression, variables that have p-value less than 0.05 in multivariable logistic regression were considered as significant.

Study participants who felt high worry about having the COVID-19 infection while showing symptoms resembled with the pandemic and who had a prison stay of 1–2 years were almost two times more prone to generalized anxiety disorder [AOR = 95% CI: 1.87 (1.05, 3.35) and 1.89 (1.01, 3.55)] as compared to those having moderately worry about COVID-19 infection and a prison stay of less than 1 year. Those having no clinical sign of insomnia, having clinically sub-threshold insomnia and having clinical moderately severe insomnia were 95, 87, and 77% less likely to have generalized anxiety disorder [AOR = 95% CI: 0.05 (0.01, 0.13), 0.13 (0.05, 0.31), and 0.23 (0.09, 0.54)], respectively, than those having clinically severe insomnia. Finally, those study participants having strong social support were 75% less likely to develop generalized anxiety disorder [AOR = 95% CI: 0.25 (0.11, 0.56)] than those having poor social support (Table 6).

## Discussion

In this study, the prevalence of major depressive and generalized anxiety symptoms were 279 (66.4%) with 95% CI of (61.4, 70.6), and 281 (66.9%) with 95% CI of (61.9, 71.9), respectively. To the knowledge of investigators’, this is the first study showing an elevated prevalence of depression and anxiety symptoms among prisoners during the COVID-19 pandemic in Ethiopia. This is supported by a previous study that showed being a prisoner as consistently related to greater symptoms of depression and anxiety (20).

The finding of high levels of depression and anxiety among prisoners in this study is in line with earlier studies conducted among prisoners in Kenya (65%), in southern Ethiopia (56.4%) and in southwest England (67.5%; 13, 30, 53). Likewise, the prevalence of major depressive disorder found in this study is in line with a study conducted among incarcerated women in the United Kingdom 61.7% (16). The prevalence found in our study is lower than found by a study conducted in Australia 80% (15) and, in New Zealand 91% (18). The reason might be differences in tools used, the sample sizes determined, and characteristics of study participants.

However, both major depressive disorder and generalized anxiety disorder were found to be higher in this study than in studies done in Jordan where 23.8% had depression while 13.1% had anxiety (14), in Chile where depression was 22% and anxiety 24% (17), in Hong Kong where depression was 19% and anxiety 14% (20), and in the United States where depression was 24.9% and anxiety 30.8% (21). Reasons for this incongruence could be differences in the study population, setting, tool used, sample size determined, and study period.

In addition, the depression and anxiety findings of this study were higher than those of pre-pandemic studies conducted among imprisoned people in Ethiopia, such as in Debre Berhan where depression was found to be 44% (31, 54), in Jimma where depression was 41.9% and anxiety 36.1% (32), in northwestern Amhara where depression was 43.8% (28), in Bahirdar where depression was 45.5% (29), and in southern Ethiopia where depression was 56.4% (30). The differences might be due to the added burden of the COVID-19 pandemic, the tools used and the sample size determined. Likewise, the prevalence of depression in this study was higher than found in studies conducted in Nigeria and south Nigeria, where it was 35 and 37%, respectively, (26, 27). The discrepancy might be due to our study being conducted during the COVID-19 pandemic, a time when there is an additional global burden, the tool used, the sample size determined, and the setting.

Moreover, the findings of our study are higher than of a study conducted on global depression, where depression was 33.7% and anxiety 31.9% (26), and among Iranian prisoners where depression was 44% and anxiety 56.3% (25). The variations in the findings might be due to the added global burden of the pandemic during our study, the tools used, the sample size determined, and the setting. Farther afield, a community-based study conducted in Canada during the pandemic revealed a lower prevalence of depression at 47.2% and anxiety 44.1% (22) than this study found. The possible reason might be that the greater number of stressors on incarcerated people leads to a higher prevalence of mental illness in this group than in the general community (13).

Of the factors associated with major depressive disorder in this study, those having no clinical sign of insomnia were 92% less likely to have a major depressive disorder [AOR = 95% CI: 0.08 (0.03, 0.21)] than those having clinically severe insomnia. Similarly, those having clinical moderately severe insomnia were 82% less likely to have a major depressive disorder [AOR = 95% CI: 0.18 (0.08, 0.42)] than those having clinically severe insomnia. The findings in this study showed that those who had moderate social support and strong social support were 51 and 64% less likely to have a major depressive disorder [AOR = 95% CI: 0.49 (0.27, 0.88) and 0.36 (0.16, 0.80)], respectively, than those having poor social support. This finding is consistent with studies conducted among prisoners in Kenya, Jimma, and Nigeria (13, 26, 32). Likewise, those having a history of diabetes mellitus were 82% less likely to have a major depressive disorder [AOR = 95% CI: 0.18 (0.04, 0.76)] than those having HIV. This finding is consistent with those of studies conducted in Jordan, Jimma, Nigeria, and southern Ethiopia (14, 26, 32). Moreover, those participants who had ever used a substance were twice as likely to have a major depressive disorder [AOR = 95% CI: 1.89 (1.07, 3.34)] as those who had never been substance users. It is difficult from this study to judge which factor is causing the other, because it is also known that depression can predispose a person to substance use habits. This is supported by the study conducted among Jimma prisoners (30, 32, 33).

In this study, factors significantly associated with generalized anxiety disorder were age, wherein those from ages 18–25 and 26–33 were 81 and 82% less likely to have generalized anxiety disorder [AOR = 95% CI: 0.19 (0.05, 0.72) and 0.18 (0.05, 0.68)], respectively, as compared to those their age ≥42. This finding is consistent with a study conducted in Jimma (32). However, being a merchant was found to increase the chance of developing generalized anxiety disorder more than two times [AOR = 95% CI: 2.08 (1.02, 4.25)] compared to being a farmer. Likewise, being a housewife increased the chance of having generalized anxiety disorder more than eight times [AOR = 95% CI: 8.51 (1.49, 48.65)] compared to being a farmer (55). In this study, those who stayed in prison for 1–2 years were almost twice as prone to generalized anxiety disorder [AOR = 95% CI: 1.87 (1.05, 3.35) and 1.89 (1.01,3.55)] than those with a stay of less than 1 year, a finding that is supported by studies conducted in Kenya and Nigeria (13, 26). Further, in this study those having strong social support were 75% less likely to develop generalized anxiety disorder [AOR = 95% CI: 0.25 (0.11, 0.56)] than those having poor social support. This is consistent with findings of studies conducted in Kenya, Jimma, and Nigeria (13, 26, 32). Moreover, those having high worry, while showing symptoms associated with COVID-19 and a long stay in the prison were almost twice as prone to generalized anxiety disorder [AOR = 95% CI: 1.87 (1.05, 3.35) and 1.89 (1.01,3.55)] than those having moderate worry and a prison stay of 1 year or less, respectively. Participants who had no clinical sign of insomnia, clinically sub-threshold insomnia or moderately severe insomnia were 95, 87, and 77% less likely to have generalized anxiety disorder [AOR = 95% CI: 0.05 (0.01, 0.13), 0.13 (0.05, 0.31), and 0.23 (0.09, 0.54)], respectively, than those who had clinically severe insomnia.

The limitation of the study fail to assess the cause and effect relationship of dependent and independent variables. Suicidal ideation and attempt not measured using standard measuring tool.

## Conclusion

The prevalence of depression and anxiety during the COVID-19 pandemic among Dessie town prisoners was found to be at least two to three times higher than found by a study done a year earlier. Factors found to be significantly associated with major depressive disorder in this study included reporting no clinical sign of insomnia, having sub-threshold insomnia, moderate social support, strong social support, diabetes mellitus, ever having used substances; whereas factors significantly associated with generalized anxiety disorder were age, occupational status of house wife or merchant, high worry while showing symptoms associated with COVID-19, length of prison stay of 1–2 years, no clinical sign of insomnia, having sub-threshold insomnia, having clinical moderately severe insomnia and having strong social support. Early screening, and treatment of depression and anxiety among imprisoned people must become a routine activity through the establishment of a mental health clinic in the prison.

## Data Availability Statement

The raw data supporting the conclusions of this article will be made available by the authors, without undue reservation.

## Ethics Statement

Ethical clearance was obtained from the Institutional Review Board (IRB) of Wollo University College of Medicine and Health Sciences, and Dessie Correctional Prison Administration, Amhara Region, Ethiopia. The approval letter has been given on the date august 10/6/2020 with reference number of CHMS/1034/2020 with five ethical committee members. The data collectors clearly explained the aims of this study to participants. Information was collected after obtaining written informed consent from each participant. The rights of study participants to refuse or discontinue participation at any time or ask anything about the study were explained. For anonymity, participants’ names were not used at the time of data collection and all other personal information was kept entirely anonymous. Confidentiality was assured throughout the study period. The method was done based on the guild line of research protocol prepared by University of Wollo and following all the procedure accordingly, and which helps us to meet the international research procedure and show the result of this study by publish on scientific journal.

## Author Contributions

MB conceived of the idea for the study, prepared the study proposal, performed the data analysis, and drafted the manuscript. MN, MT, HG, ABy, ABl, AA, ZT, KB, and MA participated through comments on the manuscript during preparation, then all co-authors read and approved the final manuscript. All authors contributed to the article and approved the submitted version.

## Conflict of Interest

The authors declare that the research was conducted in the absence of any commercial or financial relationships that could be construed as a potential conflict of interest.

## Publisher’s Note

All claims expressed in this article are solely those of the authors and do not necessarily represent those of their affiliated organizations, or those of the publisher, the editors and the reviewers. Any product that may be evaluated in this article, or claim that may be made by its manufacturer, is not guaranteed or endorsed by the publisher.

## Abbreviations

AOR: adjusted odds ratio
CI: confidence interval
COVID-19: coronavirus disease
GAD-7: generalized anxiety disorder-7
OR: odds ratio
PHEIC: Public Health Emergency of International Concern
SPSS: Statistical Package for Social Sciences
WHO: world health organization
LMICs: low- and middle-income countries.
